# ADRB2 serves as a novel biomarker and attenuates alcoholic hepatitis via the SIRT1/PGC-1α/PPARα pathway: integration of WGCNA, machine learning and experimental validation

**DOI:** 10.3389/fphar.2024.1423031

**Published:** 2024-11-21

**Authors:** Li Song, Shuo Huang, Honghao Yan, Qing Ma, Qihan Luo, Jiang Qiu, Minxia Chen, Zongyuan Li, He Jiang, Yufan Chen, Fangming Chen, Yu Du, Haozhe Fu, Lisha Zhao, Kanglu Zhao, Ping Qiu

**Affiliations:** ^1^ Tongde Hospital of Zhejiang Province affiliated to Zhejiang Chinese Medical University, Analysis and Testing Center, Zhejiang Academy of Traditional Chinese Medicine, Hangzhou, China; ^2^ School of Basic Medical Sciences, Zhejiang Chinese Medical University, Hangzhou, China; ^3^ School of Pharmaceutical Sciences, Zhejiang Chinese Medical University, Hangzhou, China; ^4^ The First School of Clinical Medicine, Zhejiang Chinese Medical University, Hangzhou, China; ^5^ Department of Medicine, Hangzhou Normal University, Hangzhou, China; ^6^ Academy of Chinese Medical Sciences, Zhejiang Chinese Medical University, Hangzhou, China; ^7^ Zhejiang Rehabilitation Medical Center, Rehabilitation Hospital Area of the Third Affiliated Hospital of Zhejiang Chinese Medical University, Hangzhou, Zhejiang, China; ^8^ School of Medicine, The Fourth Affiliated Hospital Zhejiang University, Yiwu, Zhejiang, China

**Keywords:** biomarker, alcoholic hepatitis, WGCNA, machine learning, ADRB2, SIRT1/PGC-1α/PPARα pathway, inflammation, oxidative stress

## Abstract

**Background:**

Alcoholic hepatitis is a severe inflammatory liver disease. In recent years, the incidence of AH has been on the rise, leading to an increasingly severe disease burden. Currently, there is a lack of specific biomarkers for the diagnosis and prognosis of AH in clinical practice. Therefore, the main objective of this study is to identify biomarkers closely associated with the progression of AH, to address the shortcomings in pathological diagnosis, and to identify potential therapeutic targets.

**Methods:**

Bioinformatics and machine learning methods were used to comparatively study the differentially expressed genes (DEGs) between AH patients and healthy individuals by analyzing four mRNA microarray data sets obtained from the GEO database. Subsequently, the role of potential biomarkers in AH and their mechanism of action were further confirmed by AH patients and *in vitro* and *in vivo* experiments.

**Results:**

Using differential analysis and WGCNA of the data set, a total of 167 key genes that may be related to AH were obtained. Among 167 genes, the LASSO logistic regression algorithm identified four potential biomarkers (KCNJ10, RPL21P23, ADRB2, and AC025279.1). Notably, ADRB2 showed biomarker potential in GSE28619, GSE94397, and E-MTAB-2664 datasets, and clinical liver samples. Furthermore, AH patients and *in vivo* experiments demonstrated ADRB2 inhibition and suppression of SIRT1/PPARα/PGC-1α signaling pathways, accompanied by elevated inflammatory factors and lipid deposition. *In vitro* experiments showed that ADRB2 overexpression mitigated the inhibition of the SIRT1/PPARα/PGC-1α signaling pathway, reversing the decrease in mitochondrial membrane potential, cell apoptosis, oxidative stress, and lipid deposition induced by alcohol exposure. Besides, the results also showed that ADRB2 expression in AH was negatively correlated with the levels of inflammatory factors (e.g., CCL2, CXCL8, and CXCL10).

**Conclusion:**

This study points to ADRB2 as a promising biomarker with potential diagnostic and prognostic value in clinical cohort data. In addition, in AH patients, *in vivo* and *in vitro* experiments confirmed the key role of ADRB2 in the progression of AH. These findings suggest that ADRB2 may alleviate AH by activating the SIRT1/PPARα/PGC-1α pathway. This finding provides a new perspective for the diagnosis and treatment of AH.

## 1 Introduction

The World Health Organization reports that in 2016, the number of deaths worldwide due to alcohol abuse reached three million, accounting for 5.3% of the total number of deaths (Gomez-Medina et al., 2023). Recently, as the standard of living improves and the number of individuals consuming alcohol increases, alcohol-related liver disease (ALD), especially alcoholic hepatitis (AH) has gradually evolved into a serious public health issue (Bataller et al., 2022; European Association for the Study of the Liver, 2018). AH is a severe inflammatory liver disease, and substantial evidence indicates elevated short-term and long-term morbidity and mortality rates, with a prognosis that is cause for concern. It has been reported that the average 30-day mortality rate in patients with severe alcoholic hepatitis may range from 17% to 50% (Singal and Shah, 2019; Bataller et al., 2022). Unfortunately, the current therapeutic approaches, such as corticosteroids and pentoxifylline, have limited efficacy, and new therapeutic targets and the development of safer and more effective therapeutic drugs are urgently needed. Notably, several recent studies have identified a number of biomarkers that are considered potential therapeutic targets for modulating liver inflammation pathways (McClain et al., 2021; Szabo et al., 2022).

AH is a disease type that urgently requires biomarker support. Due to the challenging nature of obtaining accurate patient alcohol consumption histories and the lack of entirely unique clinical, laboratory, or imaging features for AH, biomarkers play a crucial role in its diagnosis and management (Rutledge and Im, 2021). Recently, a study involving 114 ALD patients who underwent liver biopsy revealed that the positive predictive value (PPV) of the NIAAA-diagnosed AH criteria was 81%, with a false-negative rate of 30% (Crabb et al., 2016; Avitabile et al., 2020). Evidence suggests that only one-third of heavy drinkers will exhibit obvious clinical liver damage. This characteristic makes it challenging to predict, prevent, and tailor individualized treatment, thereby increasing the risk of progression to advanced stages of ALD in AH patients (Avila et al., 2020). Therefore, there is an urgent need to find a new and accurate method to predict or assist in the diagnosis of AH, addressing the limitations of pathological diagnosis and identifying potential therapeutic targets.

In recent years, many emerging studies have identified biomarkers that significantly correlate with the degree and severity of liver damage and inflammation by integrating sequencing technologies, machine learning, and bioinformatics (Habash et al., 2022; Niu et al., 2022). Weighted Gene Co-expression Network Analysis (WGCNA), as a systematic biology approach, plays a key role in identifying highly correlated gene clusters (modules), candidate biomarkers, and therapeutic targets. The approach contributes to predicting potential therapeutic targets and associated pathways in diseases (Larsen et al., 2023). However, there is a lack of effective non-invasive biomarkers in clinical practice to measure AH, ascertain the probability of an individual being affected by AH, and assess the severity and risk of disease progression (Avila et al., 2020). In this study, we employed bioinformatics methods and machine learning strategies to identify characteristic genes of AH and established a predictive model for AH. Furthermore, we have validated the role of potential AH biomarkers and mechanism of action in AH using *in vitro* and *in vivo* experiments, providing important insights into the diagnosis, progression, and treatment strategies of AH.

## 2 Materials and methods

### 2.1 Data collection and processing

The Gene Expression Omnibus (https://www.ncbi.nlm.nih.gov/geo/) and Array Express (www.ebi.ac.uk/arrayexpress) databases served as the sources for acquiring GSE142530, GSE28619, GSE94397, and E-MTAB-2664 datasets. Table 1 presents comprehensive information about these datasets. The “limma” program was used to background-correct, normalize, and convert the datasets to gene symbols referencing the probe annotation files for probe names. GSE142530 was used as metadata, while GSE28619, GSE94397, and E-MTAB-2664 were used to validate the diagnostic genes identified from the metadata.

**TABLE 1:** Information on microarray datasets.

Dataset	Control	Alcoholic hepatitis	Alcoholic steatosis
GSE142530	12	10	0
GSE28619	7	15	0
GSE94397	0	71	6
E-MTAB-2664	12	30	0

### 2.2 Identification and gene set enrichment analysis of DEGs

The identification of differentially expressed genes (DEGs) and subsequent Gene Set Enrichment Analysis (GSEA) were conducted utilizing the “limma” and “GSEABase” packages. Criteria for screening DEGs between the AH and control groups were adj. *p*-value <0.05 and |Log2FC| > 1.5. Expression heat maps were generated using the R package pheatmap, showing the top 30 genes exhibiting the most significant upregulation and downregulation. Furthermore, the volcano plots highlighted genes with a *p*-value <0.05 and |Log2FC| > 0.58.

### 2.3 Construction of the WGCNA co-expression network

Unsigned co-expression networks were constructed using WGCNA to detect co-expression modules. Samples were initially screened for missing values before clustering. To construct a biologically significant scale-free network, a “soft” threshold power (β) was computed based on scale-free topology criteria. Additionally, an adjacency matrix was employed to create a topological overlap matrix. A dynamic tree-cutting technique was utilized to identify gene modules. Subsequently, the network of feature genes was visualized after computing gene significance (GS), module membership (MM), and related modules with clinical characteristics. The intersection of WGCNA-derived significant module genes and DEGs was employed to identify potential gene targets associated with AH.

### 2.4 Functional enrichment analysis

The study conducted an intersection between AH-associated modules identified by WGCNA and DEGs. The shared targets underwent enrichment analysis utilizing Gene Ontology (GO), Disease Ontology (DO), and Kyoto Encyclopedia of Genes and Genomes (KEGG). Functional analysis was performed using the “clusterProfiler” package, applying a filtering threshold of *p*-value <0.05.

### 2.5 Diagnostic gene screening and diagnostic model construction

To identify relevant prognostic factors among the overlapping genes, study employed the “glmnet” package, employing the Least Absolute Shrinkage and Selection Operator (LASSO) regression analysis approach, known for its regularization and variable selection. Genes strongly associated with AH were identified using LASSO. Subsequently, a diagnostic model predicting AH occurrence was developed using logistic regression analysis and visualized as a nomogram. Receiver operating characteristic (ROC) curves were built, and the area under the curve (AUC) was calculated using the “pROC” package to evaluate the diagnostic significance of screened signature genes. Additionally, the differential expression of identified biomarkers was confirmed in the GSE142530 dataset. In addition, we analyzed the expression levels of the screened biomarkers in liver tissues in other different types of liver diseases to determine whether the potential biomarkers were AH-specific.

### 2.6 The patient sample collection

In this study, we analyzed biopsy specimens from eight patients with AH from the Fourth Affiliated Hospital of Zhejiang University School of Medicine. The inclusion criteria for AH patients are as follows: according to the 2018 EASL Clinical Practice Guidelines for the Management of Alcoholic Liver Disease, patients who actively abuse alcohol and have consumed excessive amounts of alcohol (>60 g/day) for at least 3 months prior to admission; Elevated levels of transaminase (AST>ALT, high serum levels of gamma-glutamyltranspeptidase and bilirubin, and histological diagnosis of AH are characterized by the presence of liver cell damage (hepatocyte ballooning and the presence of Mallory bodies), inflammatory infiltration (neutrophils), and peri cellular fibrosis. Inclusion and exclusion criteria for AH patients: Patients with other causes of liver disease, including chronic hepatitis B, non-alcoholic fatty liver disease, autoimmune liver disease, drug-induced liver injury, and hepatocellular carcinoma, were excluded. The control group included adjacent biopsy specimens from six patients with hepatocellular carcinoma (HCC). All study participants have obtained informed consent. This study has been approved by the Ethics Committee of the Fourth Affiliated Hospital of Zhejiang University School of Medicine (Approval No.: K2020157).

### 2.7 Immunohistochemical staining

For immunohistochemical analysis, liver samples embedded in paraffin were sectioned into 5-μm-thick slices. Antigen retrieval was performed, and endogenous peroxidase activity was blocked in the paraffin-embedded liver tissue sections. The sections were then treated with ADRB2 antibody (AF6117, Affinity), followed by washing with phosphate-buffered saline (PBS) and counterstaining with DAB (ab64238, Abcam). Digital images were captured using an optical microscope (Nikon, Tokyo, Japan) at a magnification of × 400 post-staining.

### 2.8 Disease-related gene collection and processing

The investigation into genes associated with “alcoholic liver disease” was conducted across various databases, including IPA, GeneCLIP3 (http://cismu.net/genclip3/analysis.php), MalaCards (https://www.malacards.org/), GeneCards (https://www.genecards.org/), TTD (https://db.idrblab.net/ttd/), OMIM (https://www.omim.org/), and DisGeNET (https://www.disgenet.org/). To refine the data, an average Relevance score from the GeneCards database and an average gda-score from the DisGeNET database were employed as filtering criteria. The collected information from these sources underwent UPSET analysis, pinpointing genes occurring frequently (N ≥ 3 times), possibly linked to AH. Moreover, high-frequency genes (N ≥ 3 times) from clinical datasets were recognized as potentially associated with AH. The UpSetR program was utilized to analyze DEGs from multiple AH-related clinical datasets through an UpSet plot. Subsequently, the VENNY2.1 tool (https://bioinfogp.cnb.csic.es/tools/venny/) facilitated the identification of the intersection between DEGs from the mouse liver transcriptome, ADRB2 downstream genes in the IPA database, AH-related genes database, and AH-related clinical dataset DEGs. Finally, the expression values of ADRB2 and the intersection of genes from the mouse liver RNA-Seq data and AH-related clinical chip data were extracted, followed by Spearman correlation analysis of their expression values.

### 2.9 Inflammatory factor analysis and gene correlation analysis

The investigation began by sourcing two hundred inflammatory factors from the gene set base (HALLMARK_INFLAMMATORY_RESPONSE). These factors underwent spearman correlation analysis with ADRB2, considering a *p*-value <0.05 as the criterion for filtering. The DEGs of the GSE28619 dataset were derived through differential analysis, using *p*-value <0.05 and |log2FC| > 0.58 as filtering parameters. Intersection analysis was conducted among the DEGs of GSE28619, the 200 inflammatory factors, and Spearman-genes. Subsequently, the intersecting genes underwent Protein-Protein Interaction (PPI) analysis utilizing the String database (https://cn.string-db.org/). Cytoscape (3.6.0) was employed for result visualization and refinement. Targets related to alcoholic liver disease were extracted from Geneclip3, GeneCards, DisGeNET, and IPA databases. These targets were then intersected with spearman-genes, resulting in the identification of common genes. Simultaneously, the common genes were further intersected with the DEGs of microarrays GSE28619, GSE142530, and E-MTAB-2664. Criteria for DEGs remained consistent with *p*-value <0.05 and |log2FC| > 0.58. Intersection analysis was performed, and the resulting genes underwent Spearman correlation analysis with ADRB2.

### 2.10 Establishment of an animal AH model

In the establishment of the NIAAA model, procedures adhered to previously defined protocols (Bertola et al., 2013). And 24 male C57BL/6 mice housed in a climate-controlled facility with a 12-h light/dark cycle and a temperature range of 21°C–25°C. After 1 week of adaptive feeding, the mice were randomly divided into a control group and a NIAAA group (n = 12 in each group). The NIAAA group was fed with a Lieber-DeCarli diet containing 5% (v/v) ethanol for 10 days (phase 1), while the control group mice were pair-fed with an isocaloric control diet (same calories). On day 11, ethanol-fed and pair-fed mice were gavaged with a single dose of ethanol (binge; 5 g/kg body weight) or an equal dose of maltodextrin in the early morning.

In addition, we also used the traditional Lieber-De Carli mouse model of ALD. After 1 week of adaptive feeding, another 24 mice were randomly divided into normal and ALD model groups (n = 12 in each group). The ALD group mice were fed a liquid diet containing 5% (vol/vol) ethanol (Lieber-DeCarli Ethanol Diet) for 7 weeks, while normal mice were fed an isocaloric pair-fed control liquid diet.

All animal care and procedures strictly followed the guidelines for the care and use of laboratory animals, approved by the local committee and were approved by the Institutional Animal Care and Use Committee of Zhejiang Chinese Medical University (code: IACUC-20211220-01, IACUC-20230918-15).

### 2.11 Biochemical analysis and histological assay

Serum levels of alanine transaminase (ALT), aspartate transaminase (AST), alkaline phosphatase (AKP), and lactate dehydrogenase (LDH) were assessed using an automatic biochemical analyzer (Hitachi 7020, Tokyo, Japan). Glutathione (GSH) and malondialdehyde (MDA) levels were measured using kits from Nanjing Jiancheng Bioengineering Institute (Nanjing, China) after centrifugation and separation of the supernatant. Concentrations of CCL2, CXCL8, and CXCL10 were determined using enzyme-linked immunosorbent assay (ELISA) kits from Fankewei (Shanghai, China). Liver homogenate samples underwent preparation according to commercial assay kit instructions.

After the mice were sacrificed, the liver tissues were fixed in 10% neutral-buffered formalin for histological studies. Paraffin-embedded liver tissue samples were sectioned into 4-μm-thick slices, stained with hematoxylin and eosin (H&E), imaged at × 400 magnification using an optical microscope. After embedding the frozen liver tissue in OCT compound, sections of 10 µm thickness were cut and stained with Oil Red O for histological examination of hepatic steatosis.

### 2.12 RNA-Seq and bioinformatics analysis

Mouse liver tissue was used for total RNA extraction employing TRIzol reagent following the manufacturer’s protocol. An RNA quality control method was adopted to evaluate RNA content, purity, and integrity. The workflow encompassed mRNA enrichment, cDNA amplification, end repair for blunt end generation, A-tailing, adaptor ligation, and amplification through polymerase chain reaction assay. Novogene Biotechnology Co., Ltd., conducted Illumina RNA sequencing and subsequent analysis. Differential Expressed Genes (DEGs) meeting the criteria of a *p*-value of 0.05 and a log2 (fold change) of 1.5 were chosen for further functional investigation.

### 2.13 Cell culture

The mouse hepatocyte cell line (AML12) was obtained from the ATCC collection (Manassas, United States) and cultured in a 6-well plate at 37°C in a humidified atmosphere of 5% CO2 in Dulbecco’s modified Eagle’s minimum essential medium (DMEM; Thermo Fisher Scientific, United States): F-12-Ham’s medium (GE Healthcare Life Science, United States) at a 1:1 ratio supplemented with 10% fetal bovine serum (Thermo Fisher Scientific, United States), 1:500 insulin-transferrin-selenium (Corning, United States), 40 ng/mL dexamethasone (Sigma-Aldrich, Germany), 1% nonessential amino acids (Thermo Fisher Scientific, United States), 1% amphotericin B (1000 mg/mL; Thermo Fisher Scientific, United States), 1% penicillin (1000 U/mL; Thermo Fisher Scientific) and 1% streptomycin (1000 mg/mL; Thermo Fisher Scientific, United States). This medium was removed after the AML12 cells reached 90%–100% confluence, and the cells were washed once with PBS before being given the medium free of fetal bovine serum and amphotericin B.

Human immortalized liver cell line THLE-2 was obtained from ATCC (Manassas, VA, United States). THLE-2 cells were cultured using the BEGM Bullet Kit (Lonza, Walkersville, MD, United States), according to the manufacturer’s instructions. The frozen cell suspension in a cryovial containing 1 mL was rapidly thawed in a 37°C water bath, mixed with 5 mL of culture medium, and centrifuged at 1000 rpm for 5 min. Post-centrifugation, the supernatant was discarded, and 4–6 mL of complete culture medium was added. The resulting cell suspension was cultured overnight in a flask or a 6 cm dish. Passage was performed when cell density reached 80%–90%.

### 2.14 CCK8 and biochemical indicators detection

For CCK8 detection, 10 μL CCK8 reagent was added to the culture medium 4 h before analysis. Optical density (OD) 450 values were measured using a microplate reader (Thermo Fisher Scientific, United States). In addition, the cells were harvested at the indicated times, and then the levels of ALT, AST, and LDH were detected in the culture medium. The protein concentrations of AML12 cells were measured using a bicinchoninic acid (BCA) protein assay kit (Beyotime Institute of Biotechnology, Shanghai, China). The GSH and MDA in AML12 cells were determined using commercially available assay kits, following the manufacturer’s protocol (Jiancheng Bioengineering Institute, Nanjing, China).

### 2.15 Nile red staining

After seeding in a 6-well plate, AML12 cells underwent transfection with the pCMV-ADRB2-Flag plasmid and were exposed to ethanol (100 mM) for 24 h. Subsequently, the cells were fixed in 4% paraformaldehyde for 10 min, followed by three PBS washes. Nile red solution (1 μM) was applied for 15 min at room temperature in the dark for staining. Post-staining, cells were rinsed with PBS and observed under a Zeiss Axio Observer fluorescent microscope.

### 2.16 Cell ROS detection

According to previously described research with modifications, THLE-2 cells were cultured in 6-well plates at a density of 2 × 10^5^ cells per well and exposed to 100 mM ethanol for 24 h. Cellular reactive oxygen species (ROS) levels were measured using the FITC channel of a flow cytometer (BD Biosciences, FACSuite™, United States) after treatment with the ROS Assay Kit (S0033S, Beyotime Biotechnology).

### 2.17 Detection of mitochondrial membrane potential and cell apoptosis

THLE-2 cells were seeded in a 24-well plate, transfected with the pCMV-ADRB2-Flag plasmid, and stimulated with 100 mM ethanol for 24 h. Subsequently, cells were washed with PBS and stained using MitoTracker Red CMXRos, Annexin V-FITC, and Hoechst 33,342 following the manufacturer’s protocol. Incubation was performed in the dark at room temperature for 30 min, and the cells were observed and imaged using a Zeiss fluorescence microscope.

### 2.18 Western blot analysis

Total protein from THLE-2 cells and liver tissues was isolated, separated using sodium dodecyl sulfate–polyacrylamide gel electrophoresis, and transferred onto a polyvinylidene difluoride membrane. The membrane was blocked with 5% nonfat milk at room temperature for an hour, following which primary antibodies targeting GAPDH (AC002, ABclonal), ADRB2 (AF6117, Affinity), Sirt1 (BF0189, Affinity), PGC1a (66369-1-Ig, Proteintech), and PPARα (AF5301, Affinity) were incubated overnight at 4°C. After rinsing, the membrane underwent secondary antibody incubation and was examined using an Odyssey CLx imager (LI-COR, Biosciences, Lincoln, NE). Image Studio v5.2 was employed to capture signals, and the results were normalized as fold changes relative to GAPDH expression levels.

### 2.19 Immunofluorescence staining

Liver samples embedded in paraffin were sectioned into 5-μm-thick slices. Antigen retrieval was performed, and endogenous peroxidase activity was blocked in the paraffin-embedded liver sections. Subsequently, these sections were treated with ADRB2 antibody (AF6117, Affinity) and SIRT1 antibody (BF0189, Affinity). After washing with PBS, the sections were incubated with FITC-labeled goat anti-rabbit IgG (F-2761; Invitrogen) and Cy5-labeled goat anti-mouse IgG secondary antibodies (A10524; Invitrogen). Following washing, DAPI counterstaining was performed, and digital images were collected at × 400 magnification using a fluorescence microscope (Nikon, Tokyo, Japan).

### 2.20 Statistical analysis

Statistical analysis was performed using Prism 8.0 (GraphPad Software, Inc.). Two-tailed unpaired t-tests were employed for two-group comparisons, while one-way analysis of variance was used for multiple-group comparisons. All data were presented as mean ± SEM. A *p*-value of <0.05 was considered statistically significant, while a *p*-value of <0.01 was considered highly statistically significant.

## 3 Results

### 3.1 Identification of DEGs and enrichment analysis

**FIGURE 1:**
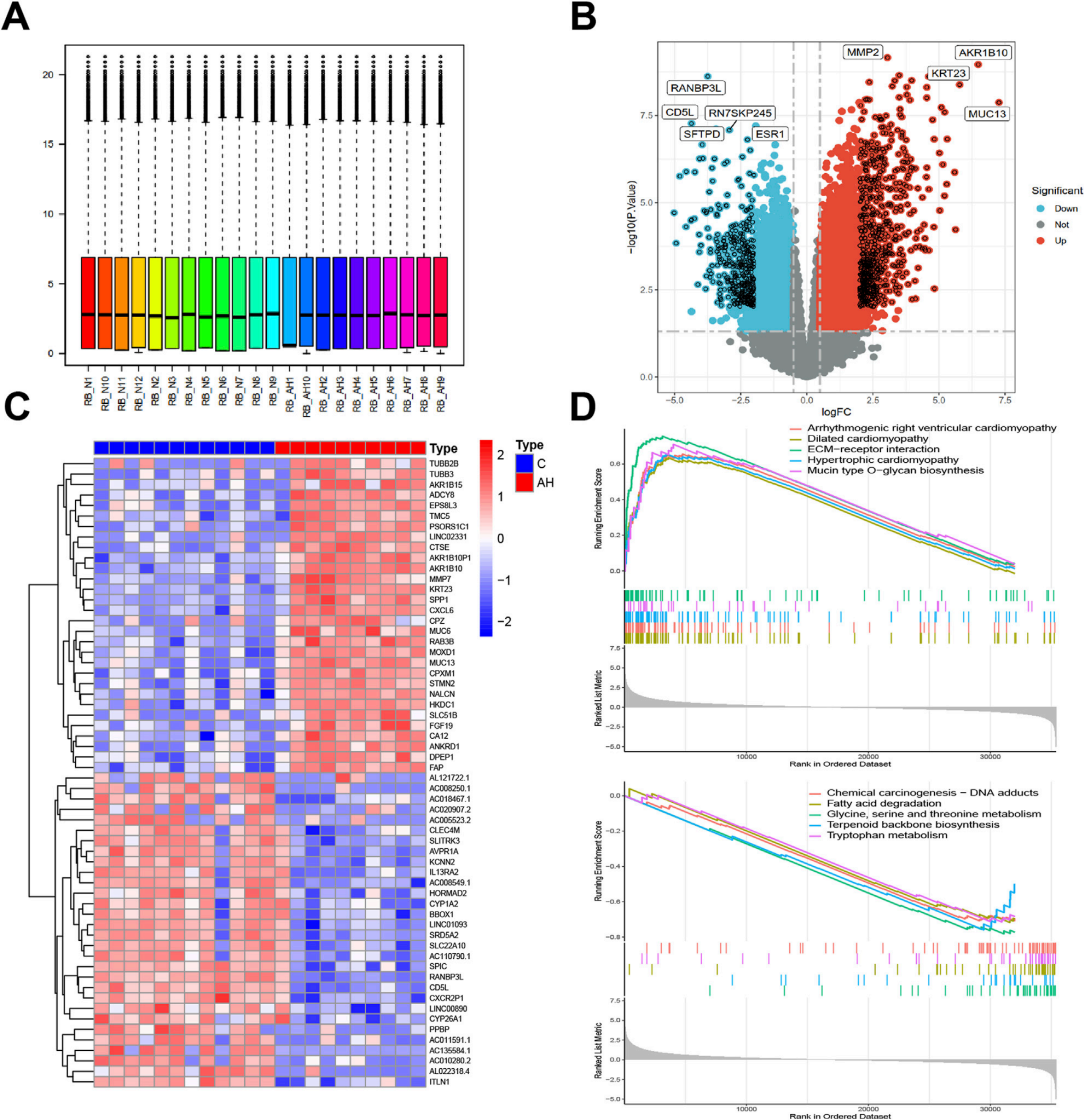
Identification of DEGs between AH liver tissues and normal samples in the metadata (GSE142530 dataset) cohort. **(A)** Box graphs showing raw data normalized across samples. **(B)** Volcano plot of the DEGs. **(C)** Heatmap of the DEGs. **(D)** Curve plot for gene set enrichment analysis of the DEGs.

Box plots were used to present normalized data, where rows represented various samples and columns depicted the gene expression levels in those samples (Figure 1A). The Volcano plot (Figure 1B) illustrates 1344 DEGs discovered, consisting of 863 upregulated and 481 downregulated genes. The heatmap (Figure 1C) displays the expression of the top 30 genes that exhibited the most significant differences between the AH and normal control groups. The GSEA results indicate that pathways related to fatty acid degradation, amino acid metabolism, cardiomyopathy, and Chemical carcinogenesis - DNA adducts in the livers of AH patients are abnormal (Figure 1D). The results show that excessive alcohol intake leads to lipid metabolism disorders in the livers of AH patients. Moreover, alcohol and its metabolite acetaldehyde can directly damage myocardial cells, causing oxidative stress, inflammation, and dysfunction in these cells, manifested as cardiac enlargement and arrhythmias (Dominguez et al., 2024). In addition, the aldehyde metabolites of alcohol can react with DNA to form DNA adducts, which may lead to cell death and the occurrence of cancer (Taniai, 2020).

### 3.2 Weighted gene co-expression network construction

**FIGURE 2:**
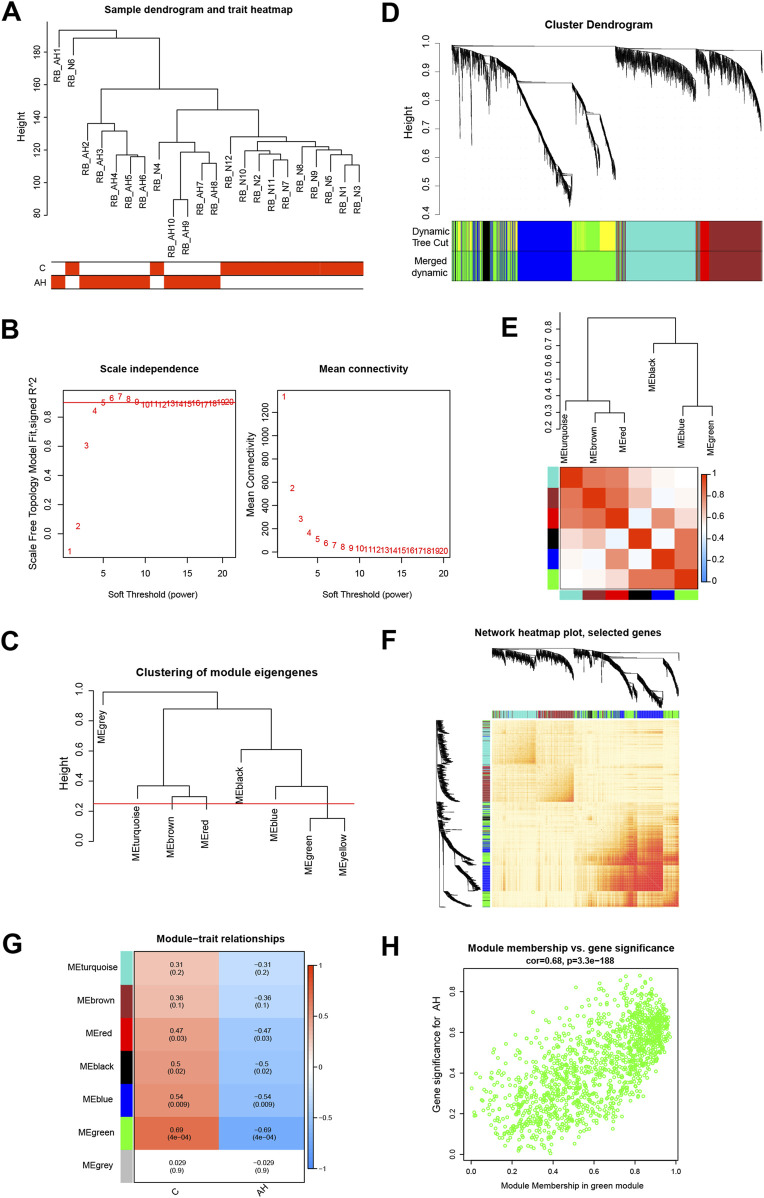
Building the co-expression network through weighted gene co-expression network analysis. **(A)** Sample clustering dendrogram where tree leaves correspond to each sample. **(B)** Analysis of the network topology for various soft-threshold powers. **(C)** A height of 0.25 was used to cut clustered dendrograms to identify and combine related modules. **(D)** Initial and combined modules beneath the tree of clustering. **(E)** Module feature genes on a collinear heat map. **(F)** Module feature gene clustering dendrogram. **(G)** Module-trait correlation heat map. Positive correlations are shown in red, and negative correlations are shown in blue. **(H)** Module membership vs gene significance scatter plot of alcoholic hepatitis.

Co-expression modules, inclusive of genes exhibiting high topological overlap similarity and co-expression levels, were formed using WGCNA. Post clustering the data via Pearson’s correlation coefficient, a sample dendrogram, alongside its corresponding trait heatmap (Figure 2A), visualizes the established sample clustering tree. Upon selecting a soft threshold of 5 (Figure 2B), given R^2^ > 0.9 and strong average connectedness, seven modules were chosen for comprehensive examination following the merging of closely related modules, constrained by a clustering height constraint of 0.25 (Figure 2C). The revised and combined modules were then depicted on the clustering tree (Figure 2D). Analyzing the correlation between modules revealed no significant associations among them (Figure 2E). Additionally, transcription correlation analysis within modules exhibited no substantial relationships, affirming the accuracy of module delineation (Figure 2F). Examining the relationship between modules and clinical symptoms revealed a negative correlation (r = −0.69, P = 4e^−04^) between the green module and AH (Figure 2G). Modules demonstrating practical significance were identified, particularly observing the strong correlation of the green module with AH in the control MM *versus* GS scatter plot (Figure 2H). Further scrutiny was conducted on all genes within the green module.

### 3.3 DEGs and functional analysis of critical module genes

**FIGURE 3:**
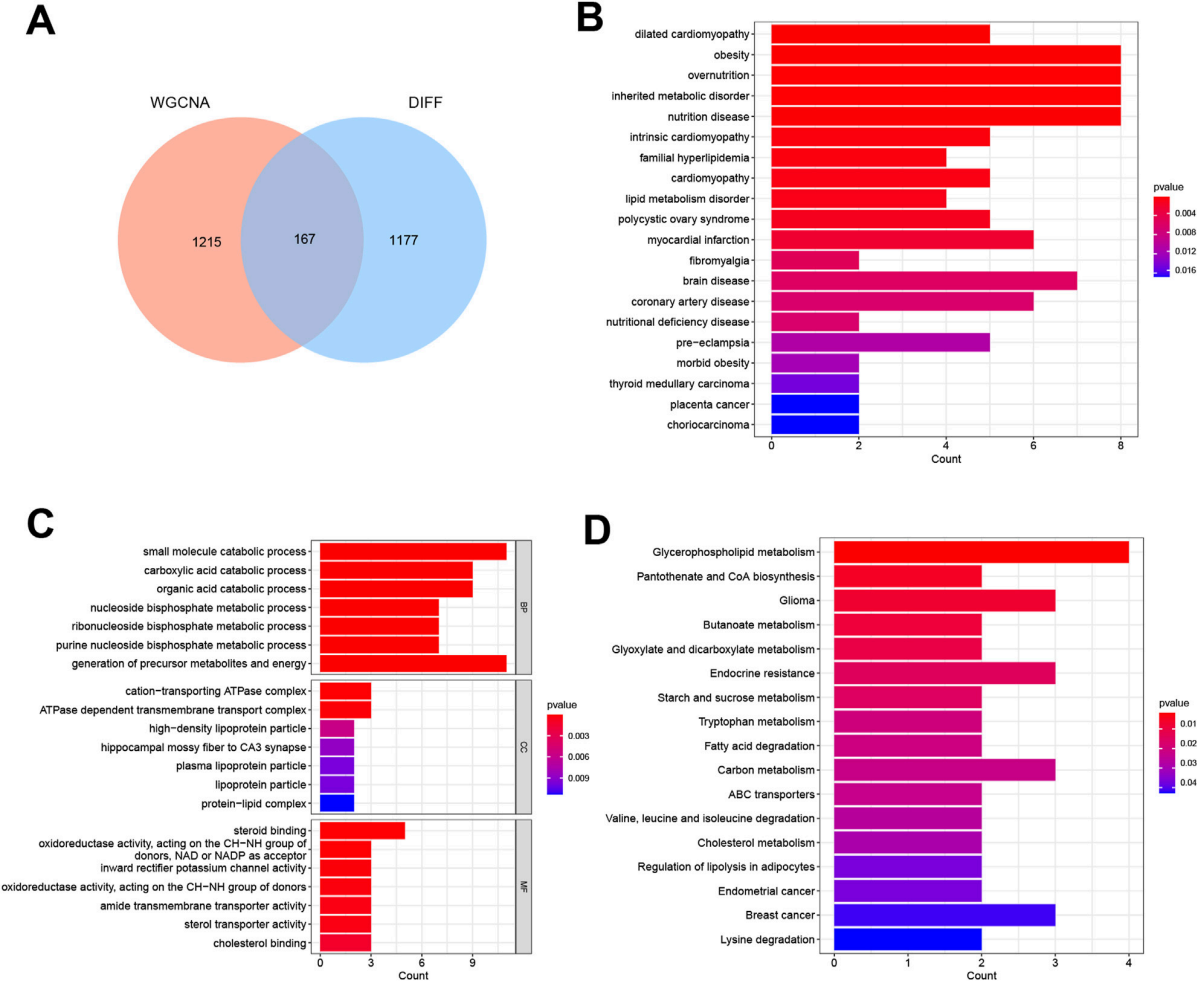
Functional analysis of DEGs combined with important module genes. **(A)** Venn diagram showing DEGs and important module genes. **(B)** Analysis of Disease Ontology. **(C)** Analysis of Gene Ontology. **(D)** Analysis of Kyoto Encyclopedia of Genes and Genomes.

By constructing a Venn diagram to overlap DEGs and key module genes, 167 overlapping genes were identified (Figure 3A). Functional analysis was performed to elucidate the biological roles of these 167 overlapping genes within the modules. Subsequent DO analysis linked these DEGs to obesity, nutrition-related diseases, hyperlipidemia, and cardiomyopathy (Figure 3B). Notably, GO enrichment analysis highlighted the concentration of Module DEGs in the production of precursor metabolites and energy, steroid binding, and small molecule catabolic processes (Figure 3C). Furthermore, KEGG enrichment analysis associated these DEGs with fatty acid degradation, cholesterol metabolism, and glycerophospholipid metabolism (Figure 3D).

### 3.4 ADRB2 were identified as a diagnostic biomarker

**FIGURE 4:**
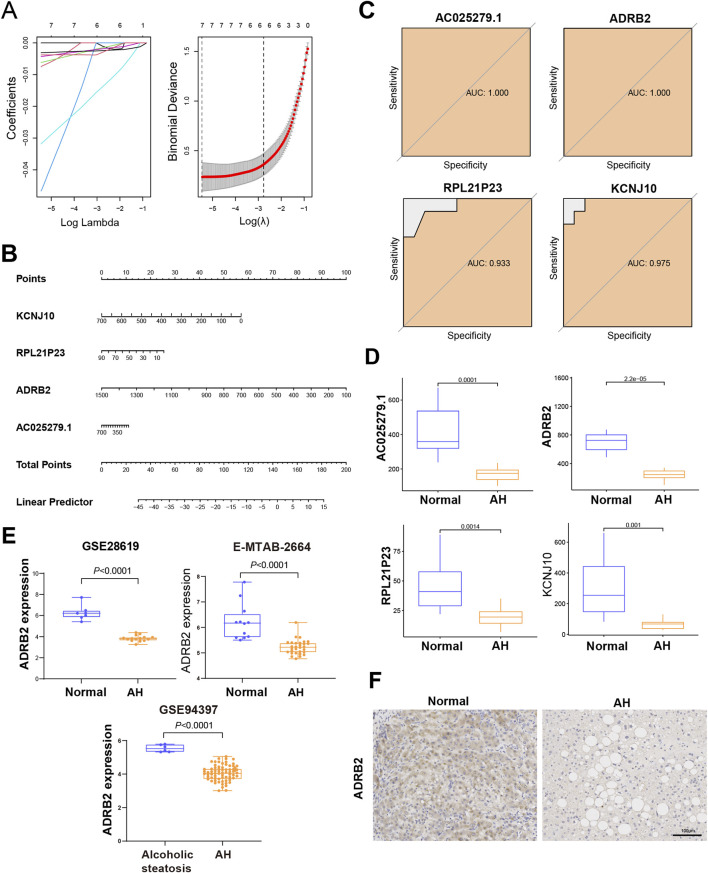
Identification and verification of the diagnostic biomarkers of alcoholic hepatitis. **(A)** From the chosen modules, diagnostic markers were screened using the Least Absolute Shrinkage and Selection Operator (LASSO) logistic regression approach. **(B)** Diagnostic nomogram. **(C)** Receiver operating characteristic curves to assess the diagnostic ability. **(D)** Box plots for the differential expression analysis in the metadata. **(E)** Box plots for the ADRB2 differential expression analysis in the testing data sets. **(F)** Immunohistochemical analysis of ADRB2 expression in normal groups and patients with alcoholic hepatitis.

Four genes—KCNJ10, RPL21P23, ADRB2, and AC025279.1—were identified as potential diagnostic biomarkers among the 167 overlapping DEGs. Using the LASSO logistic regression algorithm (Figure 4A), these genes emerged as viable candidates. A diagnostic model, represented by a nomogram (Figure 4B), was constructed via logistic regression based on the expressions of these four genes within the metadata. To validate their diagnostic value, ROC curve analysis was conducted for KCNJ10, RPL21P23, ADRB2, and AC025279.1, resulting in respective AUCs of 0.975, 0.933, 1.000, and 1.000 (Figure 4C), indicating high diagnostic potential associated with the genes KCNJ10, RPL21P23, ADRB2, and AC025279.1. Notably, their expression in the metadata was significantly lower in the AH group than in the control group, particularly evident in ADRB2 (Figure 4D). To further explore into the role of ADRB2 in AH involved the validation of its expression using GES28619, E-MTAB-2664, and GSE94397 datasets. These datasets consistently displayed reduced ADRB2 expression in AH when compared with either the control or alcoholic steatosis group (Figure 4E). Immunohistochemical staining in this study revealed a significant decrease in ADRB2 expression in liver tissues of patients with AH when compared with the control group (Figure 4F), aligning with the results obtained from bioinformatics analysis. Moreover, GEO clinical data shows that the expression of ADRB2 does not show significant changes in hepatitis B, hepatitis C, and drug-induced liver injury, suggesting that ADRB2 may be a specific biomarker for AH (Supplementary Figure S1).

### 3.5 The ADRB2/SIRT1 signaling axis was significantly suppressed in AH

**FIGURE 5:**
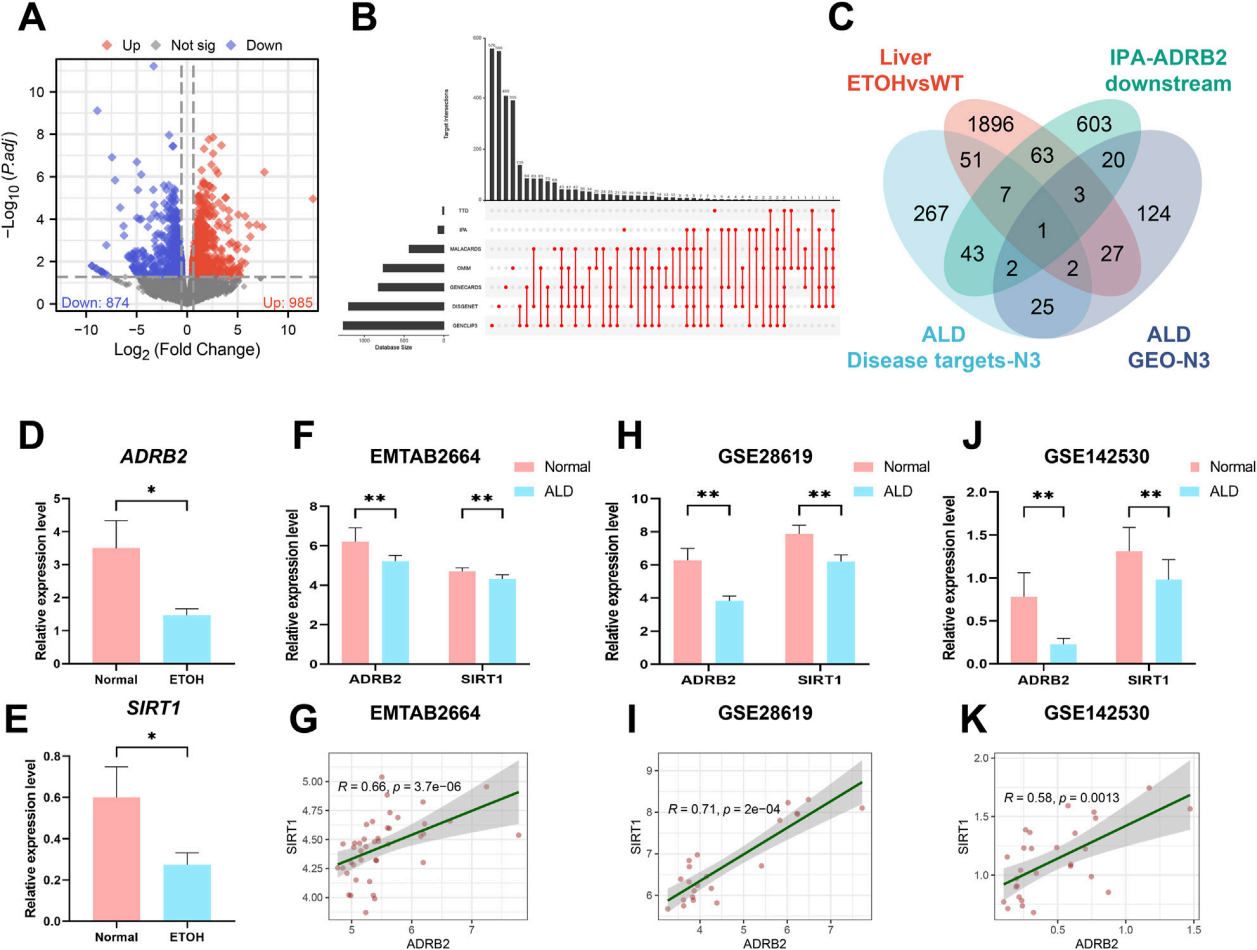
SIRT1 may be a key downstream regulatory molecule of ADRB2 in AH. **(A)** Volcano plot of mouse liver RNA-Seq (DEGs; *p* < 0.05 and log2FC > 0.58). **(B)** UpSet analysis chart of AH targets in seven disease databases. **(C)** Venn diagrams illustrate the intersection of DEGs from liver RNA-Seq, AH disease database targets with a high frequency (N ≥ 3 times), ADRB2 downstream targets from the IPA database, and AH-related targets with a high frequency (N ≥ 3 times) in GEO dataset DEGs. **(D–E)** Relative expression values of ADRB2 and SIRT1 in liver RNA-Seq data. **(F, H, J)** Relative expression values of ADRB2 and SIRT1 in the EMATB 2664, GSE28619 and GSE142530 data sets were significantly decreased in the AH group. **(G, I, K)** Spearman correlation analysis showing a positive correlation between ADRB2 and SIRT1. * Represents *p* < 0.05 and ** represents *p* < 0.01, compared with the wild-type and normal groups.

To investigate the potential processes underlying AH liver damage due to ADRB2 inhibition, RNA-Seq was employed to evaluate the gene expression profile in mouse liver tissues. DEGs between AH and Normal group mice (*p*-value <0.05 and |log2FC| > 0.58) were identified (Figure 5A). Subsequently, an UpSet plot indicated 398 targets with a frequency (N) ≥ 3 across seven disease target databases (Supplementary Table S1; Figure 5B). Venn diagram analysis suggested SIRT1 as a plausible downstream target of ADRB2 (Figure 5C). Analysis of RNA-Seq data revealed the significant downregulation of both SIRT1 and ADRB2 expression in the livers of AH group mice compared with the normal group (Figures 5D,E). Similarly, in the E-MTAB-2664, GSE28619, and GSE142530 datasets, diminished ADRB2 and SIRT1 expression was observed in the livers of patients with AH compared with the normal group (Figures 5F, H, J). Spearman correlation analysis further highlighted a significant positive correlation between ADRB2 and SIRT1 expression across the three AH datasets (Figures 5G, I, K). The above results prove that SIRT1 may be a key downstream regulatory molecule of ADRB2 in AH.

### 3.6 A negative correlation between ADRB2 and the production of inflammatory factors in AH

**FIGURE 6:**
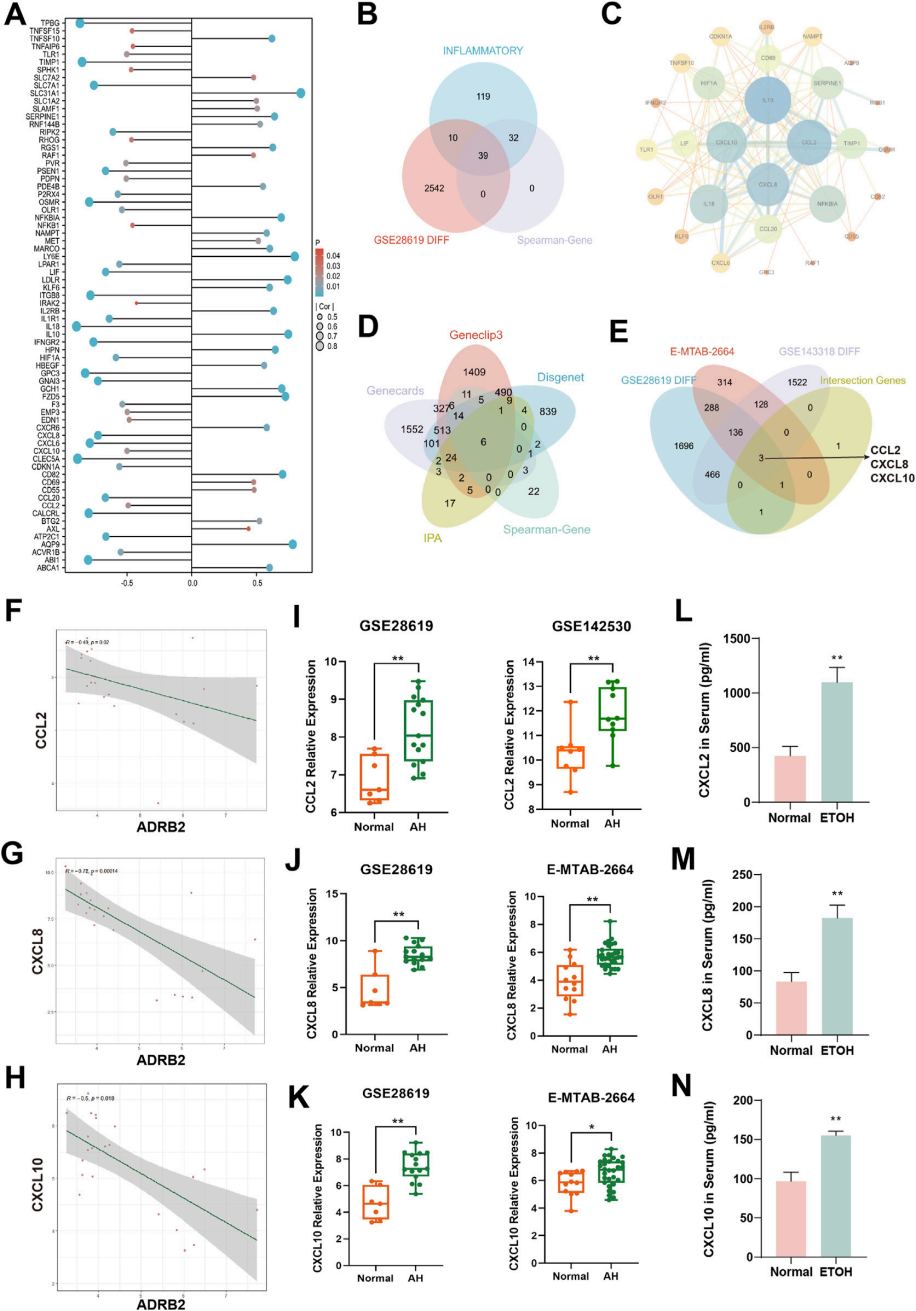
Analysis of inflammation factors and correlation with ADRB2 in alcoholic hepatitis (AH). **(A)** Differential inflammatory factors in the Spearman correlation analysis with ADRB2. **(B)** Venn diagram of inflammatory factors, Spearman-related genes, and DEGs in GSE28619. **(C)** Protein-protein interaction network of inflammatory factors intersected in graph **(B)**. **(D)** Venn diagram of AH targets from Gene CLIP3, GeneCards, DisGeNET and IPA databases and Spearman-related genes. **(E)** Venn diagram of DEGs of the microarrays GSE28619, GSE142530, and E-MTAB-2664 and the intersected genes of graph **(D)**. **(F–H)** Spearman correlation analysis of ADRB2 and CCL2 **(F)**, CXCL8 **(G)**, and CXCL10 **(H)**. **(I–K)** Gene expression levels of CCL2 **(I)**, CXCL8 **(J)**, and CXCL10 **(K)** of normal groups and patients with AH. **(L–N)** Serum CCL2 (L), CXCL8 (M), and CXCL10 **(N)** levels in mice. * Represents *p* < 0.05 and ** represents *p* < 0.01, compared with the normal or control groups.

Spearman correlation analyses among 200 inflammatory factors and ADRB2 revealed 71 differentially expressed inflammatory factors (Figure 6A). Intersection between these 200 factors and inflammatory factors from microarray GSE28619 revealed 39 shared outcomes (Figure 6B). Notably, CCL2, CXCL8, and CXCL10 exhibited higher weights in the PPI network (Figure 6C). Further intersection with alcohol-related hepatitis target genes sourced from Spearman-Gene databases (Gene CLIP3, GeneCards, DisGeNET, IPA) revealed six shared genes: CXCL8, IL10, IL1R1, CCL2, PDE4B, and CXCL10 (Figure 6D). This analysis, coupled with the exploration of DEGs from microarrays GSE28619, GSE142530, and E-MTAB-2664, led to the identification of three common genes: CCL2, CXCL8, and CXCL10 (Figure 6E). Correlation analysis between CCL2, CXCL8, CXCL10, and ADRB2 revealed significant negative correlations (Figures 6F–H, with *p*-values < 0.05 or <0.01). The results of microarray differential analysis highlighted a significant upregulation in the expression levels of these genes (CCL2, CXCL8, and CXCL10) in the liver tissues of patients with AH compared with those of normal individuals (Figures 6I–K, *p < 0.05, **p < 0.01). ELISA results from mouse serum samples further supported this conclusion (Figures 6L–N).

### 3.7 Excessive alcohol consumption leads to increased hepatic inflammation and steatosis

**FIGURE 7:**
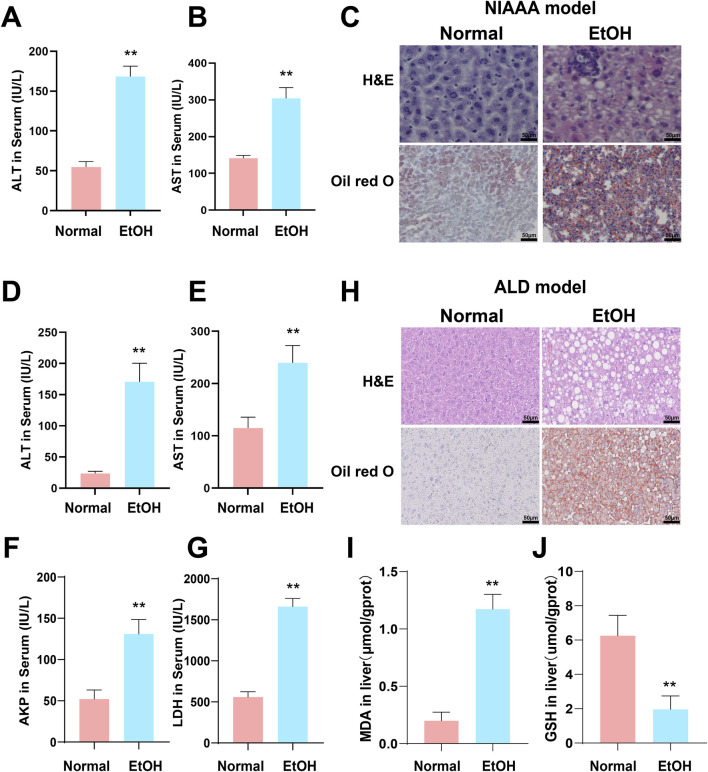
Excessive alcohol intake induces liver inflammation and oxidative stress in mice. **(A, B)** Serum ALT and AST levels of NIAAA mice. **(C)** Liver tissue sections of NIAAA mice stained with H&E and Oil Red O. **(D–G)** Serum ALT, AST, AKP and LDH levels of ALD mice. **(H)** Liver tissue sections of ALD mice stained with H&E and Oil Red O. **(I, J)** Liver MDA and GSH levels of ALD mice. * Represents *p* < 0.05 and ** represents *p* < 0.01, compared with the control group.

Compared to the normal group, serum levels of ALT and AST were significantly elevated in both the NIAAA and ALD groups (Figures 7A, B, D, E *p < 0.05, **p < 0.01). Additionally, serum levels of AKP and LDH were significantly increased in the ALD group (Figures 7F, G, *p < 0.05, **p < 0.01). H&E and Oil Red O staining revealed increased inflammatory infiltration, fat droplets, and substantial lipid deposition in the livers of NIAAA and ALD group mice compared to the normal group (Figures 7C, H). Furthermore, GSH levels in the liver tissue of ALD mice were significantly reduced, whereas MDA levels were markedly elevated, indicating oxidative stress in the liver of ALD mice (Figures 7I, J, *p < 0.05, **p < 0.01). Therefore, these data together indicate that we successfully constructed the NIAAA mouse model and ALD mouse model.

### 3.8 ADRB2/SIRT1/PGC-1α/PPARα pathway was significantly inhibited in NIAAA and ALD model

**FIGURE 8:**
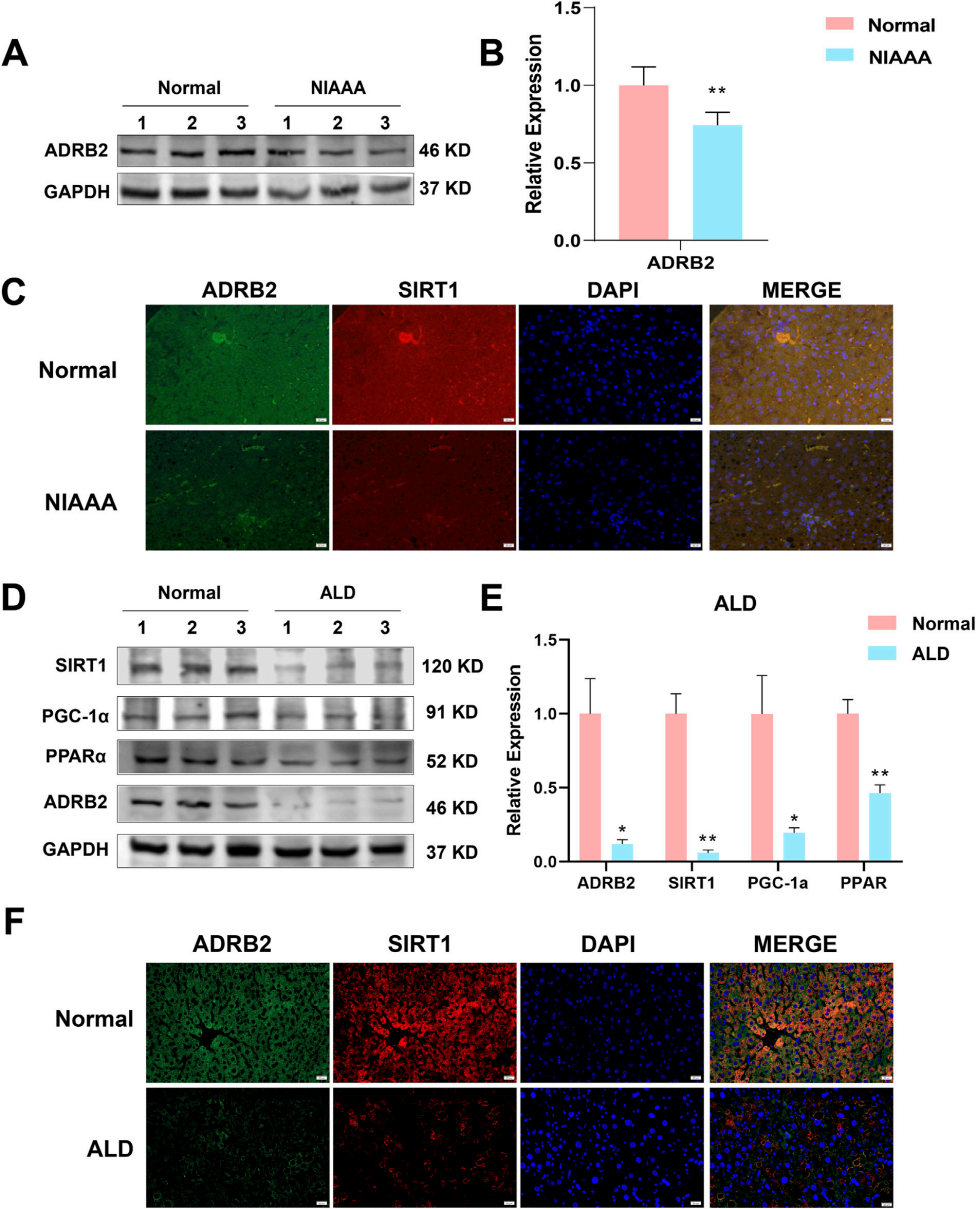
Low expression of ADRB2, SIRT1, PGC-1α, and PPARα in ethanol-induced alcoholic hepatitis. **(A)** Western blot analysis of ADRB2 in NIAAA mouse liver tissues. **(B)** Statistical results of NIAAA mouse liver western blot analysis. **(C)** Immunofluorescence of ADRB2 and SIRT1 in NIAAA mouse liver tissue. **(D)** Western blot analysis of ADRB2, PPARα, PGC-1α, and SIRT1 in ALD mouse liver tissues. **(E)** Statistical results of ALD mouse liver western blot analysis. **(F)** Immunofluorescence of ADRB2 and SIRT1 in ALD mouse liver tissue. * Represents *p* < 0.05 and ** represents *p* < 0.01, compared with the control group.

In the NIAAA model, western blot results showed that excessive drinking caused a significant decrease in the expression of ADRB2 protein in the liver of mice in the alcohol group compared with the normal group (Figures 8A, B, *p < 0.05, **p < 0.01). Additionally, the ADRB2, SIRT1, PPARα and PGC-1α protein expression in liver tissue of ALD model mice decreased significantly compared with the normal group (Figures 8D, E, *p < 0.05, **p < 0.01). Immunofluorescence results also demonstrated a noticeable reduction in the fluorescence expression of ADRB2 and SIRT1 in ALD and NIAAA group mouse livers compared with the normal group (Figures 8C, F), corroborating the western blot results.

### 3.9 ADRB2 overexpression upregulates the SIRT1/PGC-1α/PPARα pathway ameliorating ethanol-induced hepatocytes damage

**FIGURE 9:**
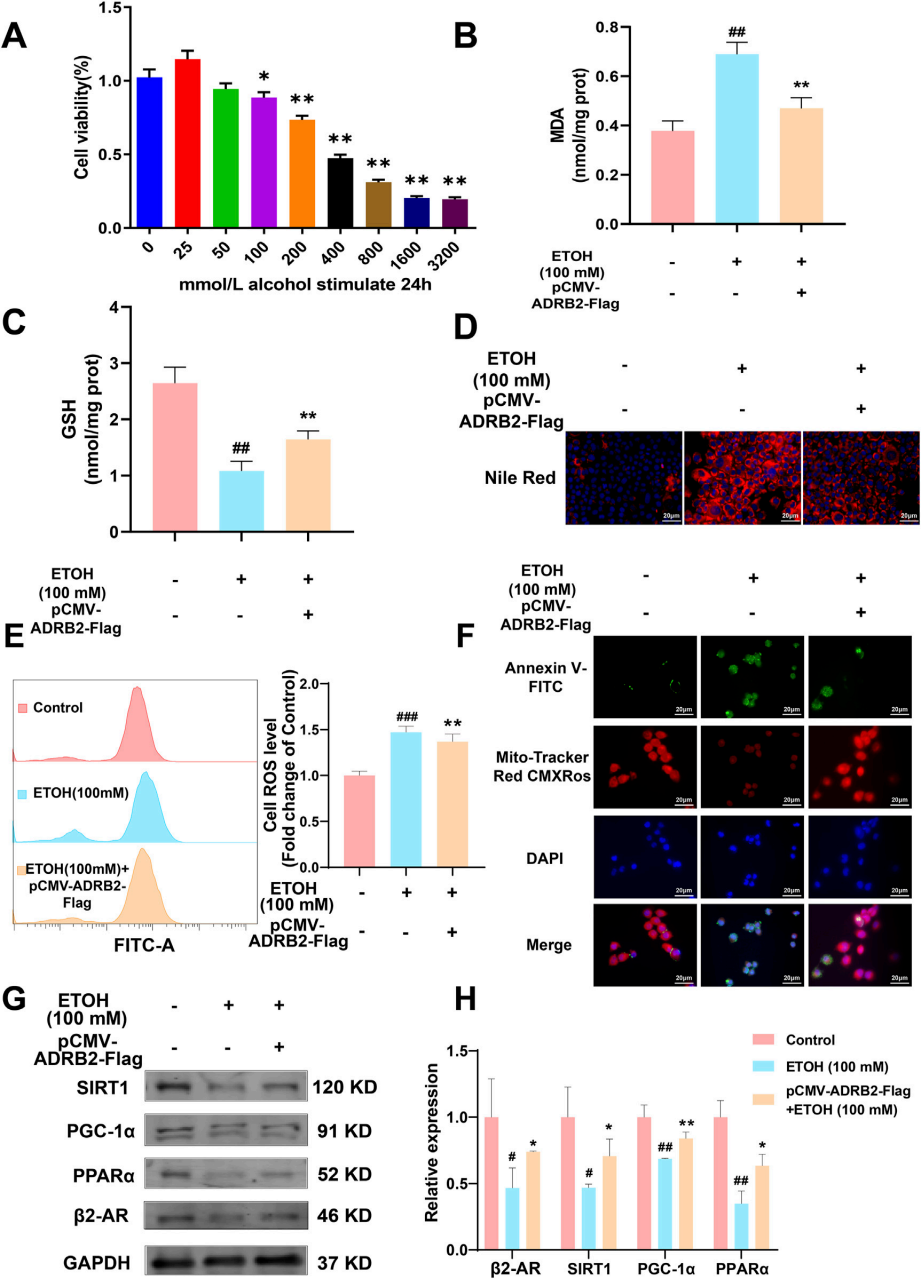
ADRB2 reverses ethanol-induced damage to the liver through the SIRT1-PGC-1α-PPARα pathway. **(A)** Cell viability of AML12 cells stimulated by alcohol at different concentrations for 24 h **(B)** MDA concentration in AML12 cells after treatment. **(C)** GSH concentration in AML12 cells after treatment. **(D)** Nile red staining of cellular lipids in AML12 cells. **(E)** Flow cytometry analysis of reactive oxygen species generation. **(F)** Mitochondrial membrane potential and detection of apoptosis of THLE-2 cells. **(G)** Western blot analysis of ADRB2, PPARα, PGC-1α, and SIRT1 in THLE-2 cells. **(H)** Statistical results of western blot analysis. # Represents *p* < 0.05 and ## represents *p* < 0.01, compared with the control group. * Represents *p* < 0.05 and ** represents *p* < 0.01, compared with the ethanol group.

*In vitro* experiments were conducted to elucidate the role of ADRB2 in protecting from ethanol-induced damage in AML12 and THLE-2 cells. The results of CCK8 assay revealed a significant decrease in the viability of AML12 cells after 24 h of stimulation with 100 mM ethanol compared to the control group (Figure 9A, *p < 0.05, **p < 0.01). Overexpression of ADRB2 markedly reduced the ethanol-induced increases in ALT, AST and LDH, MDA expression and significantly restored GSH levels (Supplementary Figure S2; Figures 9B, C, ##*p* < 0.01, **p < 0.01). In AML12 cells, nile red staining showed that ADRB2 overexpression could decrease ethanol-induced lipid deposition in liver cells (Figure 9D). Flow cytometry results further showed that ADRB2 overexpression markedly reduced oxidative stress in liver cells (Figure 9E, **p < 0.01, ###*p* < 0.001). Fluorescent staining with Mito-Tracker Red CMXRos and Annexin V-FITC demonstrated that ADRB2 overexpression significantly reversed ethanol-induced mitochondrial membrane potential decline and cell apoptosis (Figure 9F). Additionally, western blot results indicated that ADRB2 overexpression mitigated the ethanol-induced decrease in the expression of ADRB2, SIRT1, PGC-1α, and PPARα in THLE-2 cells (Figures 9G, H, *p < 0.05, **p < 0.01, #*p* < 0.05, ##*p* < 0.01). These findings suggest that ADRB2 overexpression activates the SIRT1/PGC-1α/PPARα pathway, ameliorating ethanol-induced liver cell damage, lipid deposition, and oxidative stress.

## 4 Disscussion

ALD is a liver condition caused by long-term excessive alcohol consumption, with clinical and histopathological forms ranging from initial simple steatosis to progressive steatohepatitis with accumulating fibrosis, to cirrhosis and its complications, ultimately developing into hepatocellular carcinoma. AH is a distinct clinical syndrome characterized by recent-onset jaundice in patients with continuous alcohol abuse, with or without other symptoms of liver dysfunction (Morgan et al., 2010). The underlying cause of this clinical syndrome is steatohepatitis, a disease histologically characterized by steatosis, hepatocellular ballooning, and inflammatory infiltration of polymorphonuclear neutrophils (European Association for the Study of the Liver., 2018). However, there are limitations in the diagnosis of AH, and currently, there are no effective biomarkers available for screening patients with AH for prognosis. Therefore, there is an urgent need to identify relevant biomarkers of AH and explore their mechanisms of action to provide new insights into the prevention and treatment of AH.

WGCNA is an analytical method that revolves around gene co-expression networks. Unlike unweighted gene networks, WGCNA maintains the continuous characteristics of node connections, ensuring data integrity and providing more comprehensive and robust analytical capabilities (Giotti et al., 2017; Li et al., 2018). Machine learning, rooted in statistical and computer science algorithms, is widely used for constructing predictive models and identifying biomarkers (Greener et al., 2022). Frederik et al. identified SMOC2 as a potential blood biomarker for enhancing nonalcoholic fatty liver disease (NAFLD) diagnosis through WGCNA and single-cell sequencing, offering a noninvasive alternative to liver biopsy (Larsen et al., 2023). In a separate study, Saeed et al. explored gene co-expression modules in the liver of normal individuals and those with metabolically associated fatty liver disease and related liver cancer datasets using WGCNA. They found that enhancing the expression of CGN, ZNF419, and ZNF551 or reducing SPATA20 expression might aid in maintaining liver homeostasis and improving the prognosis of liver cancer patients (Esmaili et al., 2021). Kong et al. discovered Fmo5 as a key player in apoptosis and inflammatory responses in alcoholic fatty liver disease (AFLD) through WGCNA (Kong et al., 2021). Hou et al. employed WGCNA and other systems biology approaches to pinpoint potential biomarkers associated with M1 macrophages, ferroptosis, and cuproptosis in patients with AH, including ALDOA, COL3A1, LUM, THBS2, and TIMP1(Hou et al., 2023). These studies underscore how machine learning methods have become reliable tools for identifying clinical biomarkers, holding significant potential for AH diagnosis and treatment.

This research employed a combination of WGCNA and machine learning techniques to examine the GSE28619 dataset. This analysis successfully identified four diagnostic biomarkers: KCNJ10, RPL21P23, ADRB2, and AC025279.1. Notably, the integration of literature and *in vitro* and *in vivo* experiments suggested that ADRB2 might function as a potential biomarker for AH. ADRB2 belongs to the G protein-coupled receptor family and encodes the beta-2 adrenergic receptor protein, which exhibits wide distribution in diverse tissues, including the heart, liver, and vascular smooth muscle. Increasing evidence indicates that ADRB2 plays a pivotal role in mediating oxidative stress damage and mitochondrial dysfunction in the pathogenesis and progression of diseases. Yang et al. revealed that exposure to PM2.5-induced air pollution can cause oxidative damage to myocardial cells through ADRB2 hypermethylation, whereas overexpression of ADRB2 can reverse the abnormal trend. (Yang et al., 2019).

In recent years, extensive research has confirmed the crucial role of ADRB2 in oxidative stress and inflammatory diseases, indicating that its activation can impede the progression of systemic oxidative stress and inflammatory responses (Petkevicius et al., 2021; Hasegawa et al., 2021; van Beek et al., 2023). Additionally, studies have shown not only improves aberrant lipid metabolism in the liver but also fosters hepatic regeneration via the c-met/ERK signaling cascade (Tao et al., 2019; Tao et al., 2022). Moreover, a separate investigation illustrated that genetic knockout of the ADRB2 gene exacerbates hepatic lipid accumulation in mice fed a high-fat diet (van Beek et al., 2021). Recent studies have identified that METTL14/m6A-mediated epitranscriptomic alterations disrupt ADRB signaling in adipose tissue, contributing to the development of obesity, NAFLD, and other metabolic disorders (Kang et al., 2023). Nevertheless, the precise mechanism underlying the role of ADRB2 in chronic alcohol-induced liver disease remains elusive and necessitates further exploration.

Previous research indicates that activating ADRB2 signaling not only improves aberrant lipid metabolism in the liver but also fosters hepatic regeneration via the c-met/ERK signaling cascade (Tao et al., 2019; Tao et al., 2022). Additionally, a separate investigation illustrated that genetic knockout of the ADRB2 gene exacerbates hepatic lipid accumulation in mice fed a high-fat diet (van Beek et al., 2021). Recent studies have identified that METTL14/m6A-mediated epitranscriptomic alterations disrupt ADRB signaling in adipose tissue, contributing to the development of obesity, NAFLD and other metabolic disorders (Kang et al., 2023). Nevertheless, the precise mechanism underlying the role of ADRB2 in chronic alcohol-induced liver disease remains elusive and necessitates further exploration.

In this study, our results indicated that SIRT1 might be a potential downstream target regulated by ADRB2. SIRT1, a nicotinamide adenine dinucleotide-dependent deacetylase, assumes a critical role in oxidative stress, inflammatory responses, and cellular metabolism, rendering it a promising therapeutic target for ALD (Wu Q. J. et al., 2022). Nandave et al. discovered that SIRT1 fosters mitochondrial biogenesis and activates PGC-1α and downstream Nrf-2 via the deacetylation process, thereby exerting anti-inflammatory, antioxidant, and cytoprotective effects (Nandave et al., 2023). Recent studies have indicated that modulating liver methylation and regulating the expression of J protein (MCJ) can significantly restore SIRT1 function, thereby preventing chronic alcohol-induced mitochondrial dysfunction and increased ROS production (Goikoetxea-Usandizaga et al., 2023). Zhou et al. revealed that the consumption of cyanidin-3-O-β-glucoside significantly amplifies SIRT1 activity, thereby ameliorating oxidative stress and hepatic inflammation in ALD by deacetylating NF-κB and deactivating the NLRP3 inflammasome (Zhou et al., 2020).

Emerging evidence indicates that SIRT1 may enhance PGC-1α expression, contributing to antioxidative stress mechanisms and lipid metabolism (Wu D. et al., 2022). PGC-1α, encoded by the PPARGC1A gene, functions as a transcriptional coactivator for steroid and nuclear receptors, playing a pivotal role in regulating hepatic signaling pathways and maintaining energy metabolism homeostasis. Chronic alcohol exposure suppresses the SIRT1/PGC-1α signaling cascade, disrupting hepatic lipid metabolism. Nicotinamide adenine dinucleotide (NAD+) supplements capable of activating the SIRT1/PGC-1α pathway have demonstrated efficacy in mitigating hepatic oxidative stress and lipid accumulation by bolstering NAD + levels. Both *in vivo* and *in vitro* experiments conducted in this study reveal a notable decline in SIRT1 and PGC-1α protein expression in the liver of the ethanol-exposed group, concomitant with hepatic lipid deposition and inflammatory infiltration. These findings suggest that SIRT1 and PGC-1α could serve as crucial downstream targets of ADRB2 in mitigating chronic alcohol-induced liver injury.

PPARα, a pivotal downstream regulatory factor in lipid metabolism modulated by SIRT1, has garnered significant attention. As a component of the nuclear receptor family, PPARα, along with its companions β, δ, and γ, plays a critical role as a transcriptional regulator in lipid transport, fatty acid oxidation, and inflammation (Xiao et al., 2017; Wang M. et al., 2019). Notably, PPARα is predominantly expressed in hepatic tissue. Both *in vitro* and *in vivo* investigations have demonstrated that alcohol abuse markedly impedes the SIRT1/PPARα pathway, leading to diminished NAD + supply and resulting in mitochondrial dysfunction and suppressed β-oxidation of fatty acids within the liver. Recent studies underscore that deficiencies in PPARα exacerbate hepatic lipid accumulation and exacerbate liver injury in ALD (Zhang et al., 2023). Importantly, the activation of PPARα has increasingly been recognized as an effective therapeutic approach for ALD. Research by Ni et al. has revealed that dietary supplementation with prickly pear seed oil can diminish ROS generation and enhance fatty acid oxidation by upregulating PPARα/PGC-1α, thereby ameliorating liver damage and lipid deposition induced by oxidative stress (Ni et al., 2022). Another study has shown that dietary supplementation with astaxanthin can mitigate liver oxidative stress and inflammation by activating the Nrf2 and PPARα signaling pathways, thereby mitigating chemical injury to hepatic cells. In our investigation, we observed a significant reduction in the expression of SIRT1 and PPARα in the liver under conditions of excessive alcohol exposure. This inhibition correlates positively with liver damage induced by oxidative stress and lipid accumulation. These findings suggest that dysregulation of the ADRB2/SIRT1/PGC-1α/PPARα signaling pathway may have a pivotal role in the progression of ALD, especially in AH.

In addition, our study identified that ADRB2 exerts negative regulation on various cytokines through the integration of bioinformatics analysis, as well as *in vivo* and *in vitro* experiments. Numerous studies have confirmed that chronic alcohol consumption promotes the release of pro-inflammatory cytokines such as C-C motif chemokine ligand 2 (CCL2/MCP-1), leading to the recruitment of monocytes and exacerbating the progression of AH (Duryee et al., 2022; Georgakis et al., 2022). However, Ambade et al. discovered that Cenicriviroc alleviated inflammation in ALD mice by blocking the CCL2-mediated signaling pathway (Ambade et al., 2019). Numerous pieces of evidence indicate a significant elevation of C-X-C motif chemokine ligand 8 (CXCL8/IL-8) in patients with ALD cirrhosis, which prompts Kupffer cell activation and leads to hepatic inflammation infiltration (Martin-Gonzalez et al., 2022). Recent studies have revealed that neutralizing CXCL8 or specifically blocking CXCR1/2 (CXCL8 receptors) could be a promising therapeutic strategy for treating ALD (Wieser et al., 2017; Nishimura et al., 2021). Furthermore, an increasing body of evidence suggests that C-X-C motif chemokine ligand 10 (CXCL10/IP-10) plays a crucial role in the progression of ALD (Liang et al., 2019). Dai et al. found that inhibiting CXCL10 could disrupt the interaction between Kupffer cells and liver sinusoidal endothelial cells, thereby reducing hepatic stellate cell activation and hepatocyte necroinflammation (Dai et al., 2020). Additionally, Wang et al. propose that modulating the MED23-CCL5/CXCL10 axis could be a potential approach for clinically intervening in liver fibrosis (Wang Z. et al., 2019). These findings collectively suggest that ADRB2 may play a critical role in combating AH inflammatory responses by inhibiting the expression of CCL2, CXCL8, and CXCL10.

## 5 Conclusion

**FIGURE 10:**
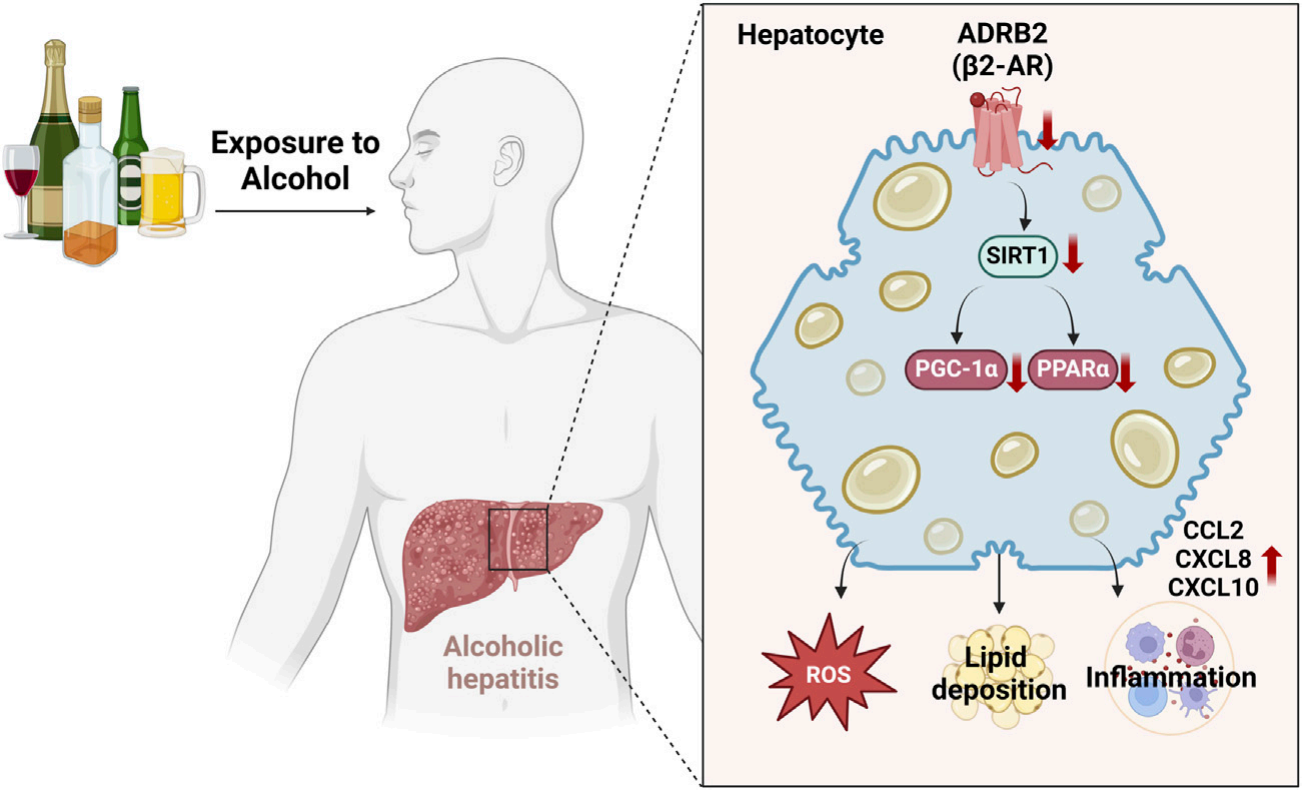
Schematic diagram illustrating the mechanism by which excessive alcohol intake inhibits the ADRB2-SIRT1/PGC-1α/PPARα signaling pathway and aggravates alcoholic hepatitis.

In this study, we used weighted gene co-expression network analysis (WGCNA) and machine learning techniques to discover that ADRB2 may serve as a potential biomarker for AH. Additionally, the results of AH patients, NIAAA, and ALD mouse models have indicated that excessive alcohol consumption inhibits the ADRB2/SIRT1/PGC-1α/PPARα pathway, leading to elevated levels of oxidative stress and cytokines such as CCL2, CXCL8, and CXCL10, accompanied by hepatic steatosis (Figure 10). Furthermore, similar results were observed in in vitro cell experiments, whereas overexpression of ADRB2 could reverse alcohol-induced oxidative stress damage and lipid deposition in hepatocytes. These findings suggest that ADRB2 may represent a valuable and promising target for the diagnosis and treatment of alcoholic liver disease.

## Data Availability

The original contributions presented in the study are included in the article/Supplementary Material, further inquiries can be directed to the corresponding authors.
