# Supplementary figures and images for: ADRB2 serves as a novel biomarker and attenuates alcoholic hepatitis via the SIRT1/PGC-1α/PPARα pathway: integration of WGCNA, machine learning and experimental validation
Source: Front Pharmacol. 2024 Nov 21;15:1423031. doi: 10.3389/fphar.2024.1423031 (PMC11617210; doi:10.3389/fphar.2024.1423031)

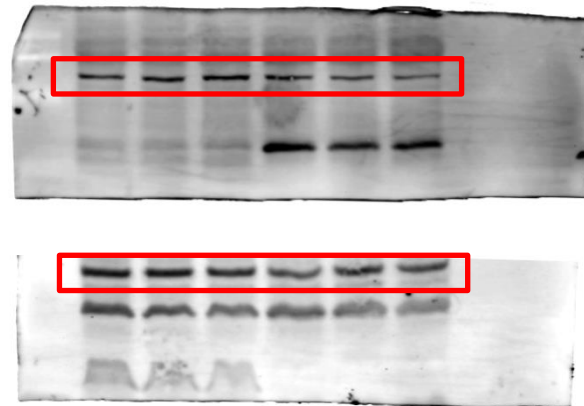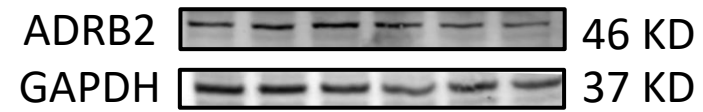

Supplement: Supplementary file 1 [file DataSheet2.PDF]

WB raw data

Fig 8A

A

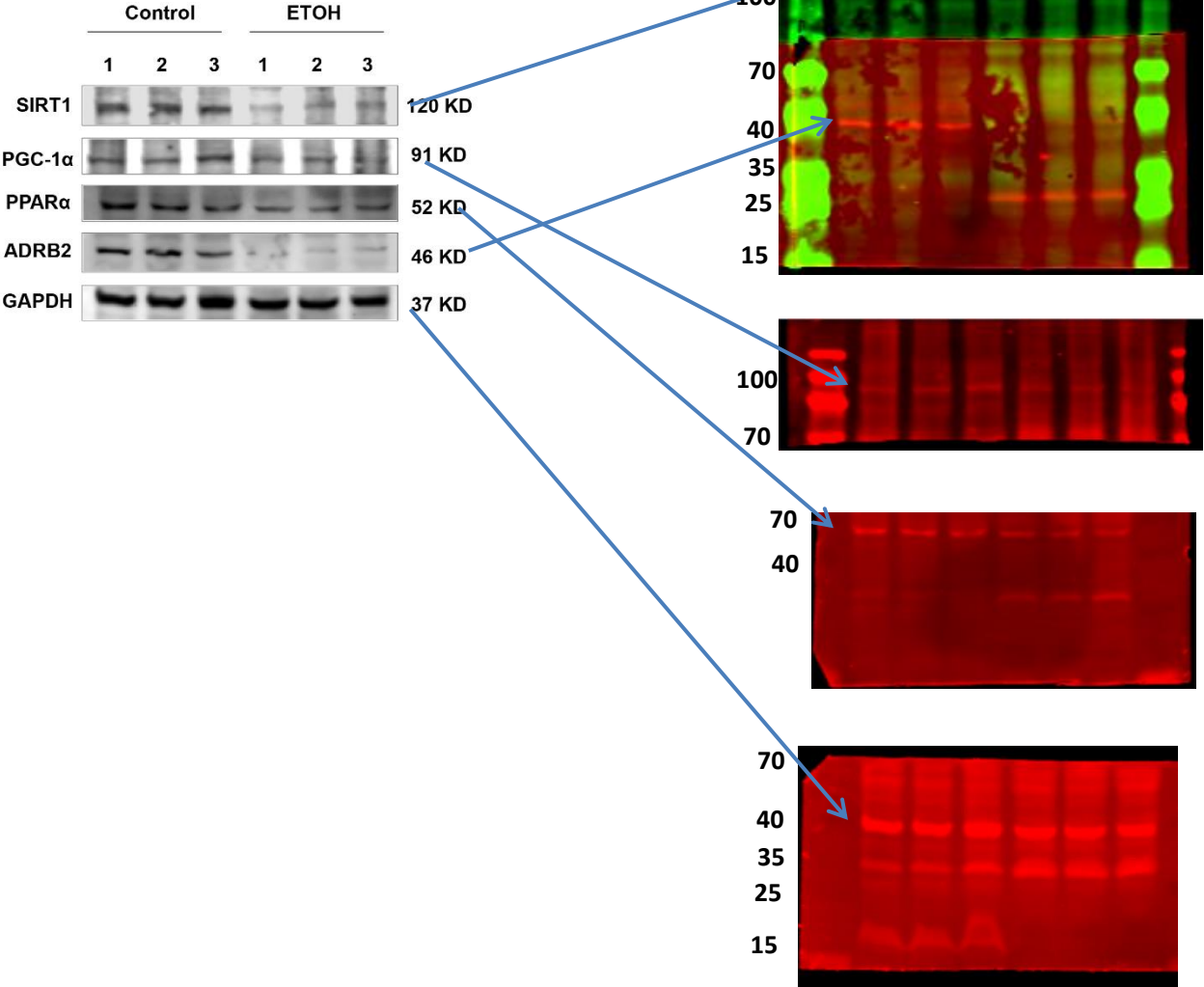

Fig 9G

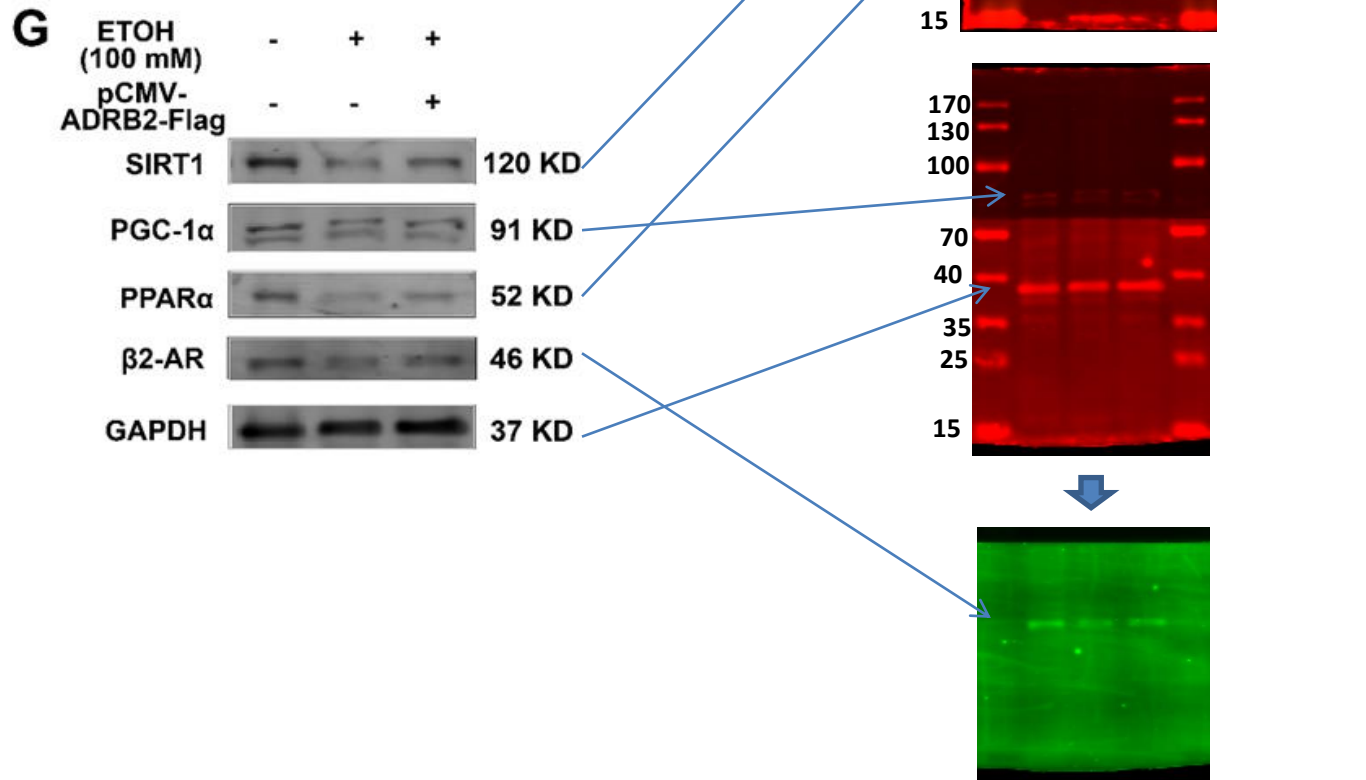

Supplement: Supplementary file 2 [file DataSheet1.PDF]
